# Supplementary material for: Genetic variation in IL-4 activated tissue resident macrophages determines strain-specific synergistic responses to LPS epigenetically
Source: Nat Commun. 2025 Jan 25;16:1030. doi: 10.1038/s41467-025-56379-8 (PMC11762786; doi:10.1038/s41467-025-56379-8)
Supplement: Supplementary file 1 — Supplementary Information [file 41467_2025_56379_MOESM1_ESM.pdf]

Supplementary Fig. 1

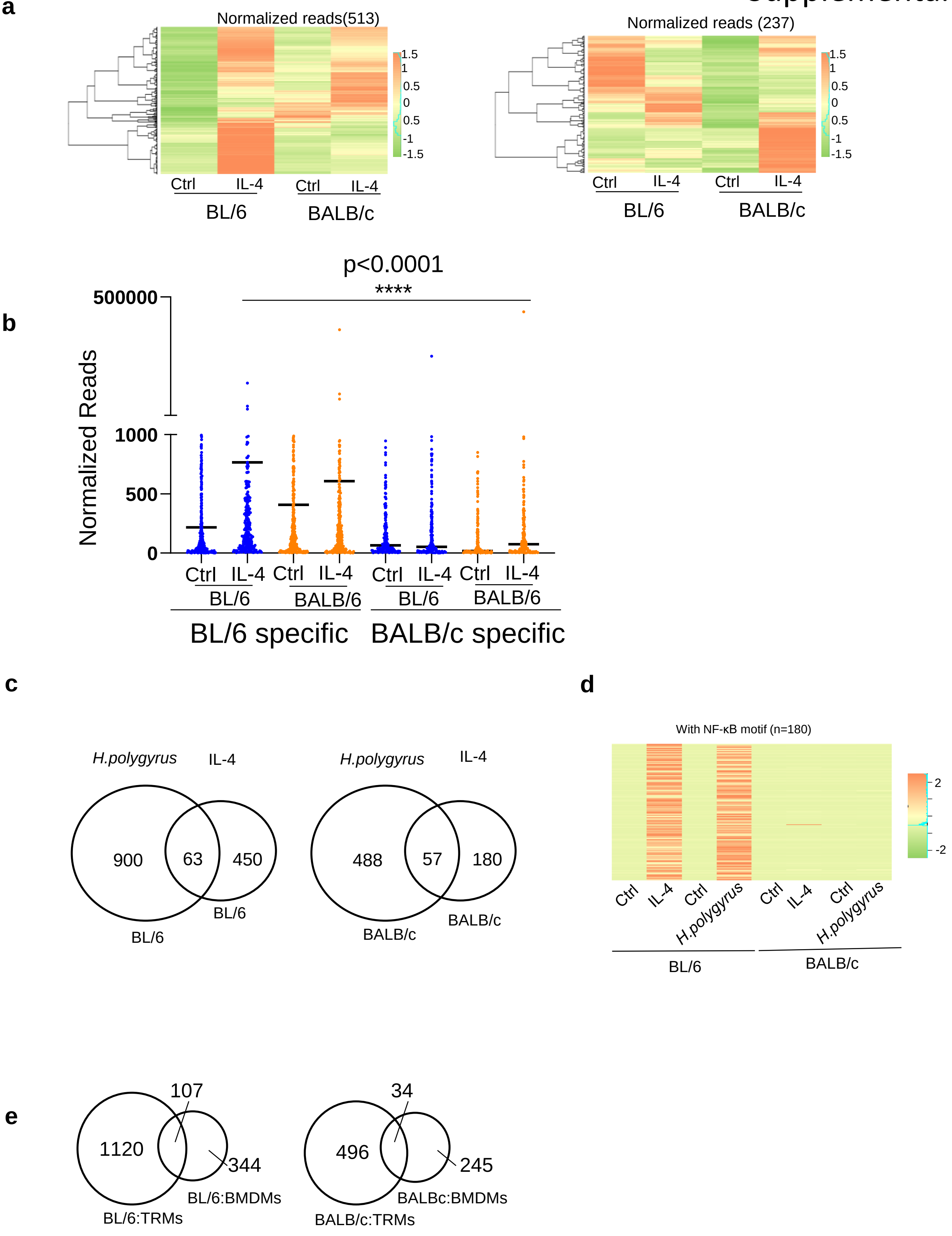

**Supplementary Fig. 1. Characteristics of strain-specific upregulated genes after IL-4 treatment.**

(a) A heatmap displays normalized reads for genes (from Fig. 1g) that are significantly induced (FDR ≤ 0.05 and ≥2-foldchange compared to Ctrl) as determined by DESeq2 analysis. Each row represents the z-score of the normalized reads.

(b) Scatter plots displaying the RNA expression levels of strain-specific induced genes (513 in BL/6 and 237 in BALB/c mice. p-values were calculated using an unpaired t-test, two-sided.

(c) The Venn diagram shows the overlap of genes significantly induced (FDR ≤ 0.05, ≥2-fold) identified by the EBSeq method in peritoneal TRMs after IL-4 treatment or in peritoneal TRMs from H. polygyrus-infected BL/6 and BALB/c mice.

(d) A heatmap displays foldchange compared with Ctrl for genes (from Fig. 4c) with the NF-κB motif either in the promoter region (-400 bp to +100 bp from TSS) or in promoter-distal regions defined by chromatin loops from Hi-C (n=180). Each row represents the z-score of fold change compared to Ctrl.

(e) Venn diagram illustrating the overlap of genes significantly induced (FDR ≤ 0.05) identified by the EBSeq method in peritoneal tissue resident memory T cells (TRMs) and bone marrow-derived macrophages (BMDMs) following IL-4 treatment in Bl/6 and BALB/c mice.

Supplementary Fig. 2

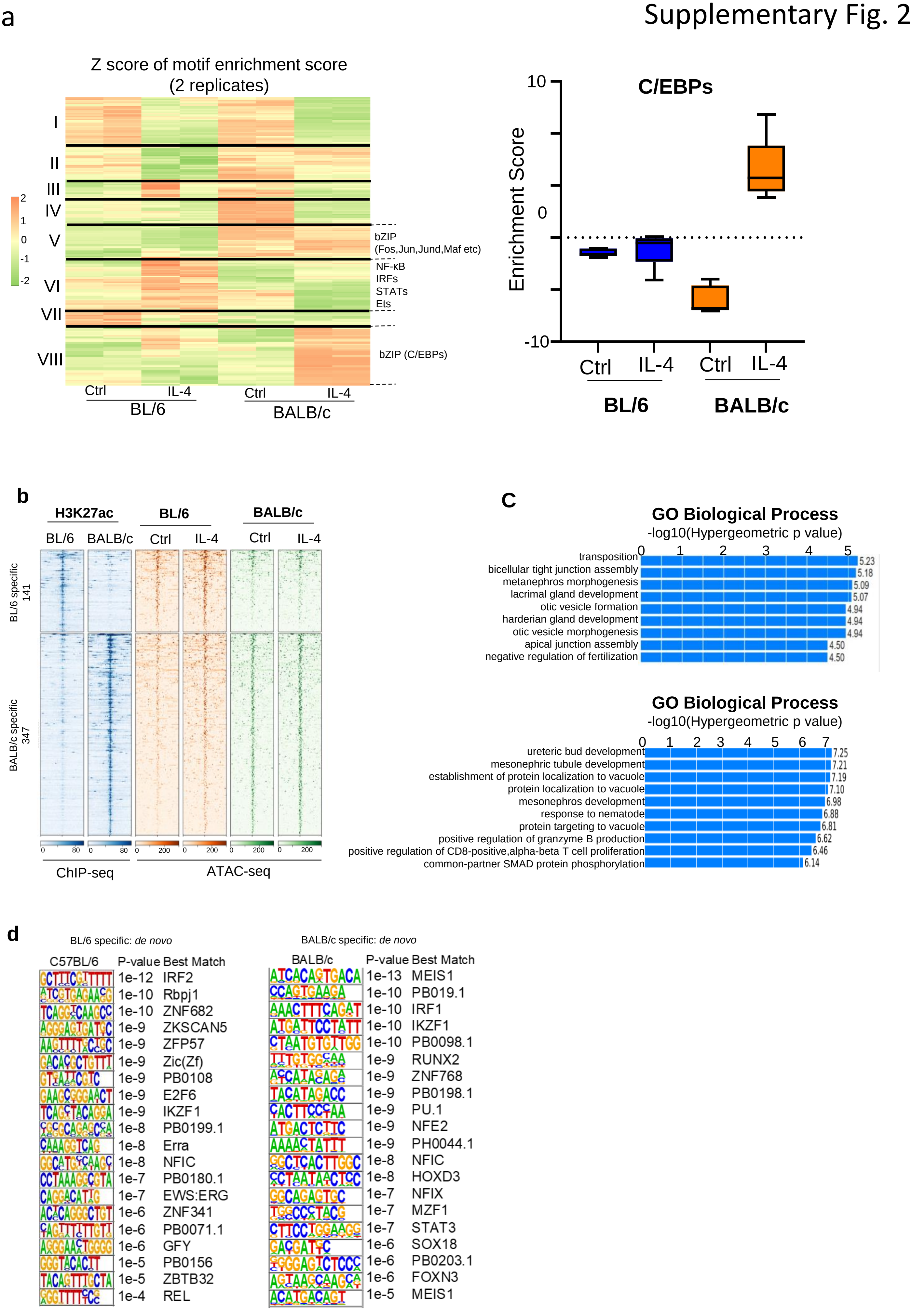

**Supplementary Fig. 2. The motif analysis identifies motifs that are functionally associated with chromatin open sites and enhancer regions in response to IL-4 treatment.**

(a) (Left) The heatmap displays the frequencies of transcription-factor motifs in ATAC-seq data, which were assessed via ChromVAR and separated by hierarchical clustering. (Right) The transcription-factor motif frequencies in ATAC-seq data for a select motif (C/EBP), which exhibits differential enrichment between BL/6 and BALB/c as assessed via ChromVAR. Each motif value represents the average of motif enrichment scores of the two replicates. Data are presented as mean value of motif enrichment scores from all motifs +/- SEM.

(b) Heatmaps, generated using deepTools (refer to the Materials and Methods section), were employed to visualize the ChIP-seq data for histone (H3K27ac) and ATAC-seq data specifically at strain-specific enhancer regions identified through BEDTools intersect analysis. The number of strain-specific enhancers is also displayed.

(c) Enrichment analysis of strain-specific enhancer regions based on biological process Gene Ontology (GO) terms was conducted using the Genomic Regions Enrichment of Annotations Tool (GREAT). The GREAT algorithm (http://great.stanford.edu/) was employed for this analysis. The values on the x-axis represent the -log10 of the Hypergeometric p-value in the pathway analysis.

(d) Motif analysis was performed using HOMER within the peaks of the strain-specific enhancers. The top 20 *de novo* motif are showed

Supplementary Fig. 3

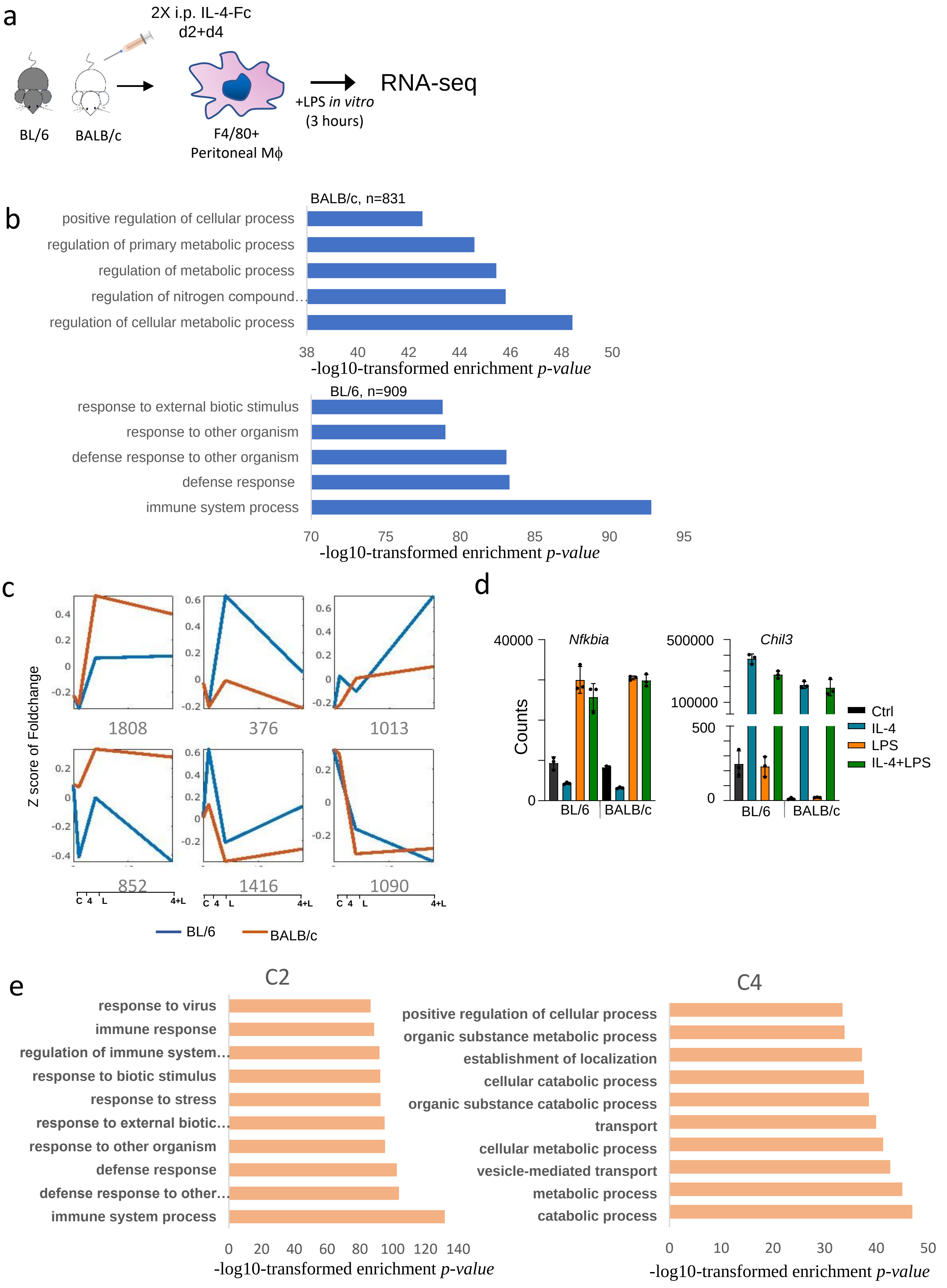

f

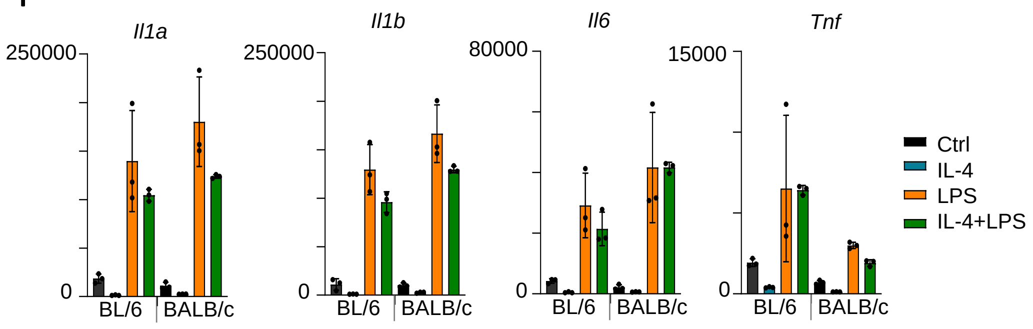


g

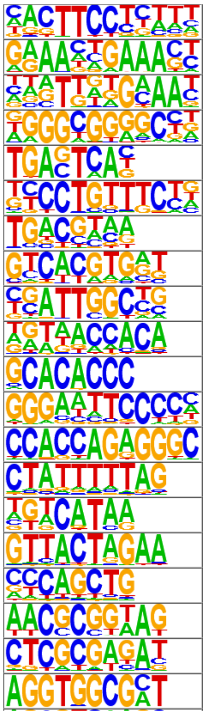

| C57BL/6 P-value | Best Match |
|---|---|
| 1e-610 | Sfpi1 |
| 1e-177 | IRF3 |
| 1e-127 | CEBPA |
| 1e-118 | KLF1 |
| 1e-117 | JunB |
| 1e-64 | PRDM4 |
| 1e-51 | FOSL1:JUND |
| 1e-43 | TFEB |
| 1e-37 | NFY(CCAAT) |
| 1e-29 | RUNX-AML |
| 1e-26 | EKLF |
| 1e-24 | NFkB-p65 |
| 1e-23 | BORIS |
| 1e-19 | MEF2A |
| 1e-16 | NFIL3 |
| 1e-12 | Bcl6b |
| 1e-10 | Ap4 |
| 1e-10 | ELF4 |
| 1e-9 | GFX |
| 1e-9 | PB0117.1 |

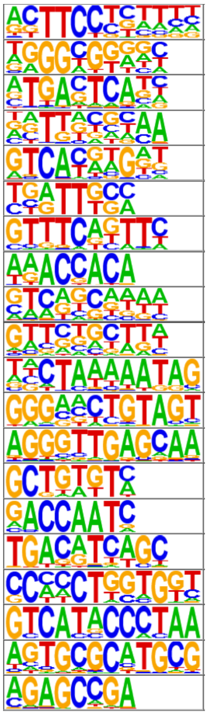

| BALB/c P-value | Best Match |
|---|---|
| 1e-418 | Sfpi1 |
| 1e-112 | KLF1 |
| 1e-96 | BATF |
| 1e-81 | NFIL3 |
| 1e-54 | MITF |
| 1e-48 | Dux |
| 1e-48 | IRF3 |
| 1e-26 | RUNX |
| 1e-23 | Mafb |
| 1e-23 | ZNF449 |
| 1e-22 | MEF2D |
| 1e-20 | GFY |
| 1e-19 | CEBPD |
| 1e-18 | BACH1 |
| 1e-17 | NFYA |
| 1e-16 | CREB1 |
| 1e-14 | BORIS |
| 1e-12 | PB0059 |
| 1e-10 | NRF |
| 1e-8 | POL010.1 |

**Supplementary Fig. 3. Characteristics of the synergistic activation of genes in TRMs.**

(a) Overview of experimental design for IL-4 primed macrophages *in vivo* and LPS treatment *in vitro* for 3 hours.

(b) Functional enrichment of predicted biological processes in BALB/c-specific upregulated genes (LPS vs Ctrl, n=831) and the BL/6-specific upregulated genes (IL-4+LPS vs LPS, n=909), as shown in Fig.3b. The x-axis represents the -log10-transformed enrichment p-value.

(c) Differentially expressed genes were identified by DESeq2 (FDR≤0.05) that were changed at any treatment between strains. The differential genes then were further separated into 6 kinetic patterns by *k-means* clustering based on the foldchange compared with Ctrl. Numbers of genes in each cluster are shown. Activation is noted on the x-axis, where 'C' indicates Ctrl, ‘4' indicates IL-4 treatment, 'L' indicates LPS treatment, and ‘4+L' indicates IL-4 and LPS cotreatment.

(d) Bar graph showed the RNA-seq counts on the selected genes after IL4 or LPS treatment (n = 3).

(e) Functional enrichment of predicted biological processes in C2 and C4, as shown in Fig. 3c. The x-axis represents the -log10-transformed enrichment *p-value*.

(f) Bar graph showed the RNA-seq counts on the selected genes after IL4 or LPS treatment (n = 3).

(g) Full list motif result of Fig. 3g. The top 20 *de novo* motif are showed

# Supplementary Fig. 4

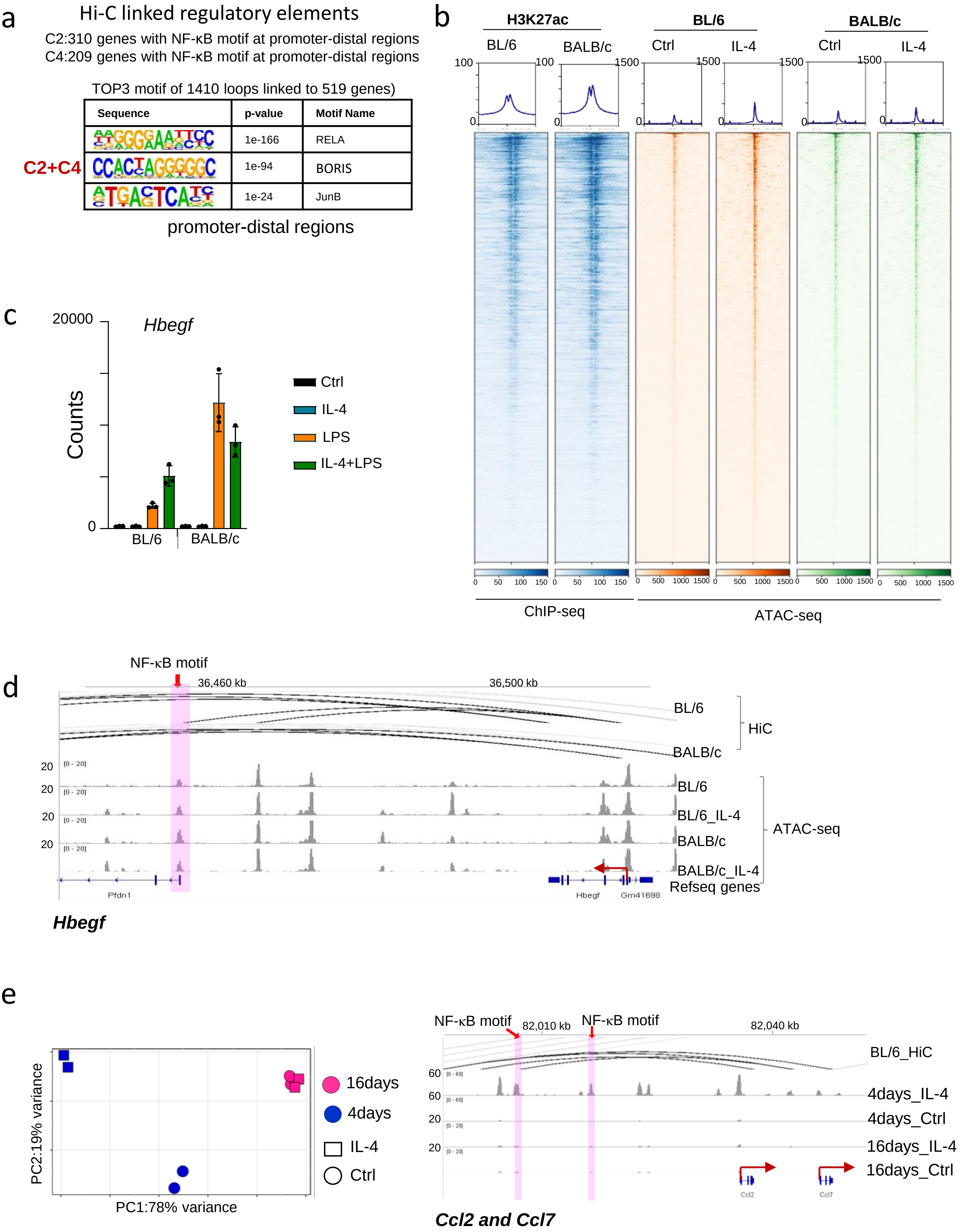

**Supplementary Fig. 4. Characteristics of chromatin structure related to the synergistic activation of genes in TRMs from BL/6 mice.**

(a) Motif analysis by HOMER revealed enrichment of the NF-κB motif in the genes whose promoter-distal regions, as defined by Hi-C, contain the NF-κB motif defined by MEME program in BL/6 mice. The number of genes in C2 and C4 with NF-κB motifs in promoter-distal regions, analyzed by MEME, was indicated at the top.

(b) A heatmap displays the intensity of H3K27ac ChIP-seq and ATAC-seq signals in the promoter-distal regions of genes from the C2 and C4 gene lists after IL-4 stimulation in TRMs from two strains. The density of peaks in the ATAC-seq data indicates that the majority of peaks are induced in the promoter-distal regions of gens from C2 and C4 (from Fig. 3c).

(c) Bar graph showed the RNA-seq counts on the selected synergistic response gene (*Hbegf*) from C2 cluster in Fig. 3c after IL-4 +LPS treatment (n = 3).

(d) Genome browser tracks (IGV) illustrate examples of promoter-distal regulatory element enriched with NF-κB motifs linked to the promoter region of *Hbegf* gene. The pink bar represent ATAC-seq peaks around the chromatin loop region from Hi-C containing NF-κB motif.

(e) Principal component analysis (PCA) of ATAC-seq data from IL-4 treatment at days 4 and 16 (n = 2 per group) shows that after prolonged IL-4 treatment, the cell state returns to the naïve state in BL/6 mice (left). Genome browser tracks (IGV) illustrate examples of regulatory elements enriched with NF-κB motifs linked to the promoter regions of the Ccl2 and Ccl7 genes, which are no longer accessible after 16 days of IL-4 treatment. The pink bars represent ATAC-seq peaks containing NF-κB motifs (right).

# Supplementary Fig. 5

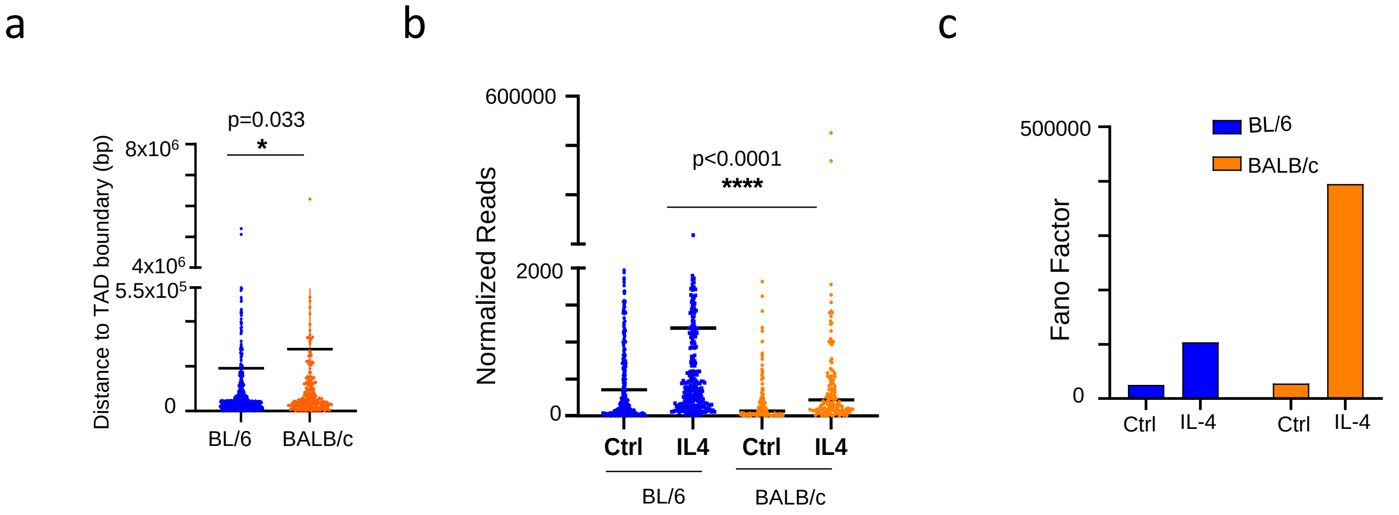

**Supplementary Fig. 5. Characteristics of genes induced specifically in different strains.**

Similar to Fig. 5c, 5d, and 5e, this figure focuses on the strain-specific induced genes identified by DESeq2 in Fig. 1f. Specifically, we selected genes meeting the threshold of FDR ≤ 0.05 and having more than a 2-fold change compared with Ctrl in both strains (513 in BL/6 and 237 in BALB/c mice).

(a ) The dot plots showing the distance of the promoter regions of these strain-specific induced genes to the TAD boundary regions. p-values were calculated using an unpaired t-test, two-sided. Data are presented as mean ± standard deviation.

(b) Scatter plot display the expression levels of the strain-specific induced genes (≥2-foldchange) (513 in BL/6 and 237 in BALB/c mice).

(c) The bar plot shows the FANO factor value of these genes.

# Supplementary Fig. 6

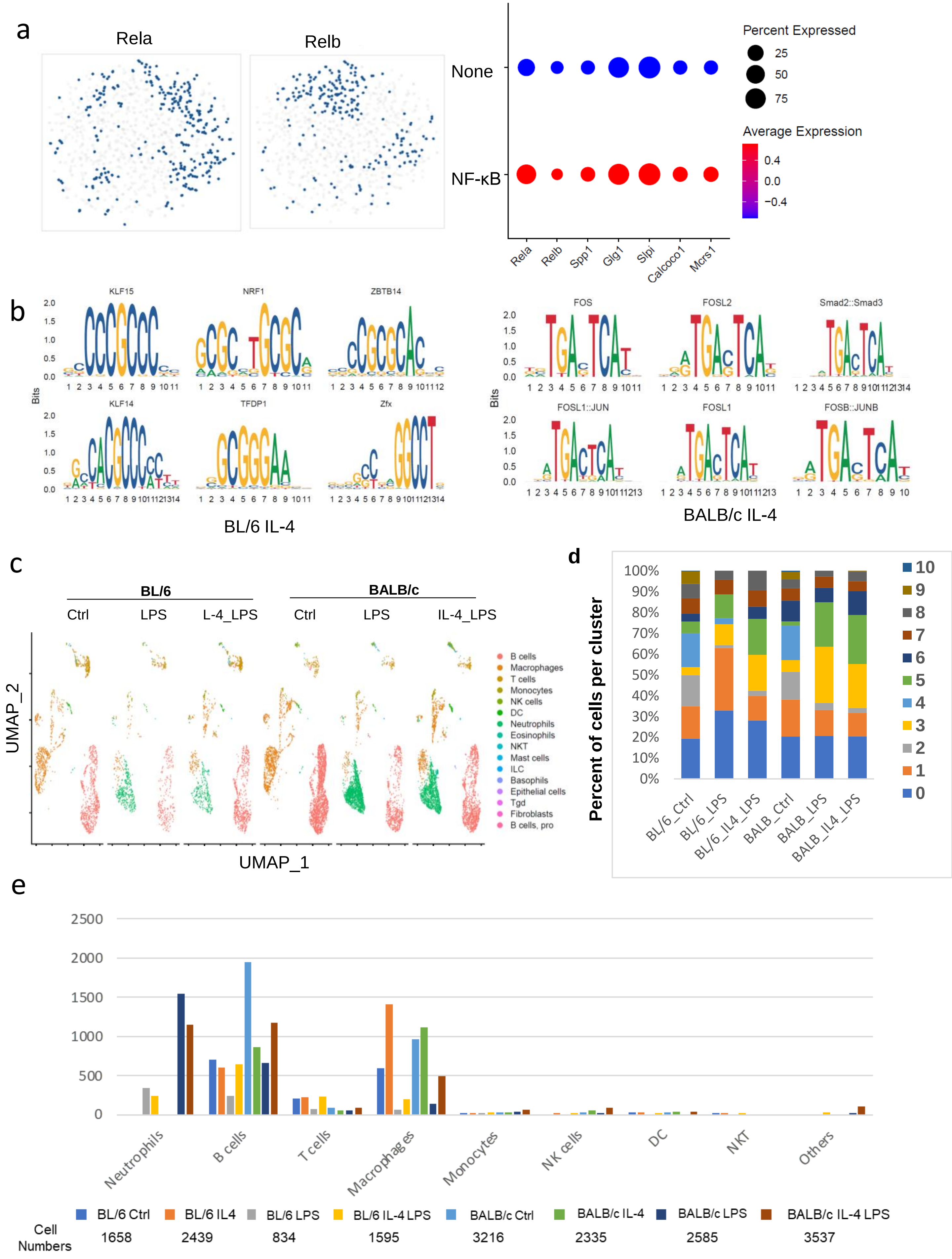

f

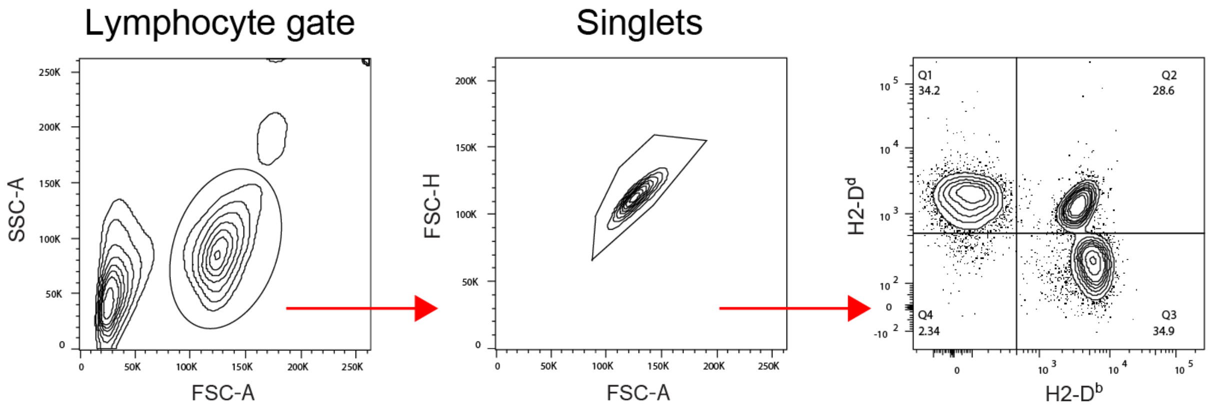

Lymphocyte gate
Singlets
SSC-A
FSC-A
FSC-H
FSC-A
H2-D^d
H2-D^b
Q1
34.2
Q2
28.6
Q3
34.9
Q4
2.34

**Supplementary Fig. 6. The features at the single-cell level of TRMs after treatment with IL-4 or LPS.**
(a) UMAP representation of regulon distribution from SCENIC analysis results, illustrating the distribution of the Rela and Relb regulons in individual cells. Each point represents a single cell, colored according to its assigned regulon, highlighting distinct cell populations identified within the dataset (left). The dot plot shows the expression levels of key genes involved in NF-κB regulation across cell clusters. The size of each dot corresponds to the percentage of cells expressing the gene, while the color intensity indicates the average expression level within that cluster (right).
(b) Enrichment analysis of the top 6 motifs in TRMs after IL-4 treatment from both BL/6 and BALB/c mice, conducted using chromVAR as part of the single-cell ATAC-seq dataset analysis.
(c) UMAP plot was created to visualize changes in the clusters of peritoneal cells derived from scRNA-seq data in chimeric mice. The clusters were color-coded according to their annotated cell types.
(d) Histogram displays the cell type composition determined by single-cell analysis. We utilized the 'FindVariableFeatures' function with 'nfeatures = 50' to visualize changes in cluster composition among samples. The results indicate a decrease in cluster 2 after LPS treatment and an increase in cluster 6 after IL-4+LPS treatment compared to LPS treatment alone.
(e) Bar plot illustrating the number of cells across various cell types in different groups. The values displayed beneath graph represent the total cell count for each group.
(f) Gating strategy for FACS-analysis of blood samples from bone marrow reconstituted F1 recipients.

# Supplementary Fig. 7

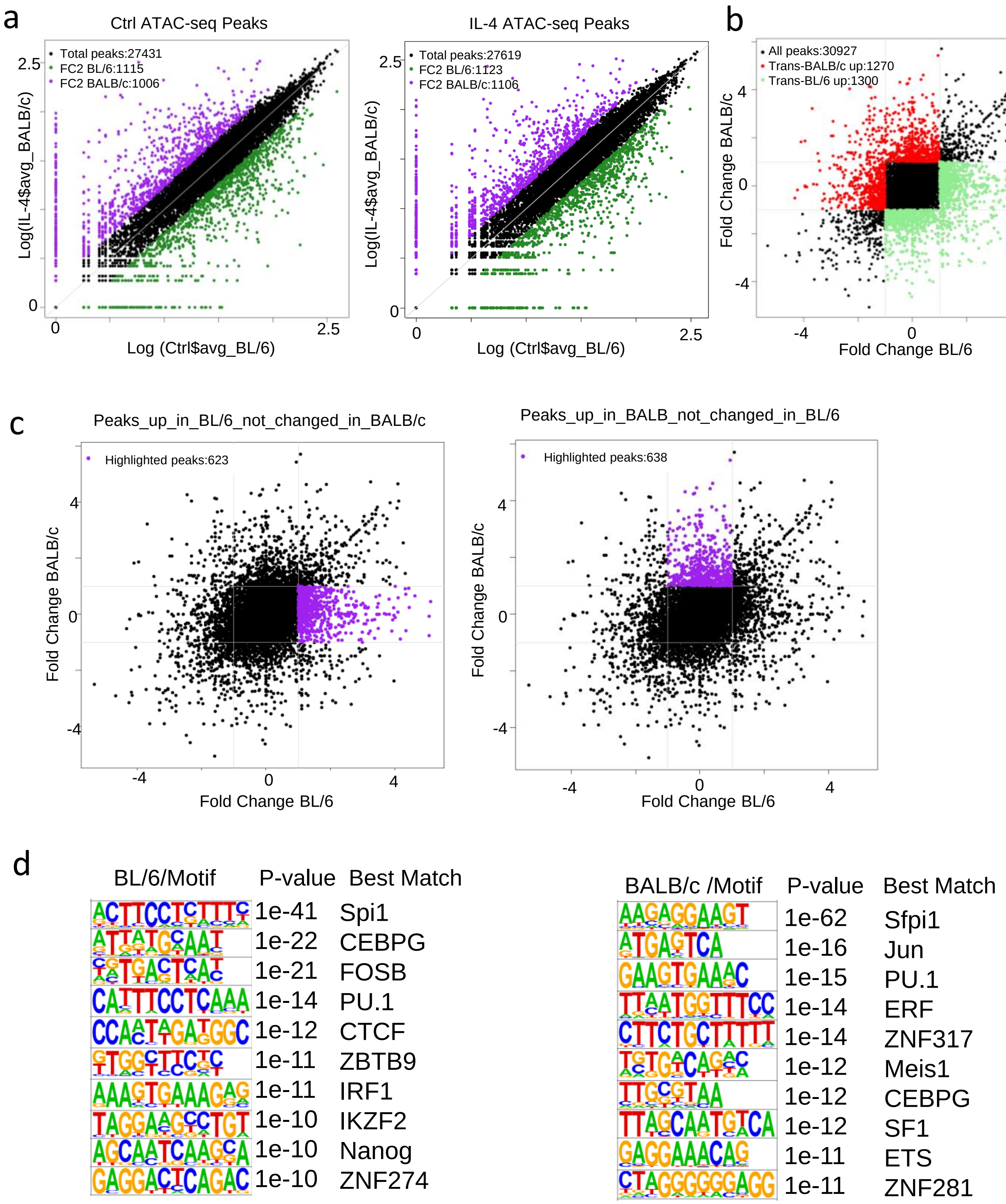

**Supplementary Fig. 7. ATAC-seq analysis of F1 mice determines BALB/c and BL/6 allele specific responses to IL-4 within the same peritoneal macrophage.**

(a) Scatterplots showing BALB/c and BL/6 specific peaks by differential analysis showing more than 2 fold change in F4/80+ peritoneal macrophages from Ctrl naïve untreated F1 mice and IL-4-Fc treated F1 mice.

(b) Ratio/ratio plot showing the fold change between Ctrl and IL-4 activated peaks from the BL/6 allele with the fold change between Ctrl and IL-4 activated peaks from the BL/6 allele. In red are shown the BALB/c specific peaks that are altered by IL-4 and in green are the BL/6 specific peaks that are altered by IL-4.

(c) Scatterplots were created to visualize the changes in peaks compared to the Ctrl for peritoneal cells derived from ATAC-seq data in F1 mice. Purple dots indicate the peaks that changed in their annotated allele but showed no change in the other allele.

(d) Motif result of Supplementary Fig. 7c. The top 10 *de novo* motif are showed
