# Supplementary material for: Genetic variation in IL-4 activated tissue resident macrophages determines strain-specific synergistic responses to LPS epigenetically
Source: Nat Commun. 2025 Jan 25;16:1030. doi: 10.1038/s41467-025-56379-8 (PMC11762786; doi:10.1038/s41467-025-56379-8)
Supplement: Supplementary file 3 — Reporting Summary [file 41467_2025_56379_MOESM3_ESM.pdf]

nature portfolio

Corresponding author(s): P'ng Loke

Last updated by author(s): Dec 11, 2024

Reporting Summary

Nature Portfolio wishes to improve the reproducibility of the work that we publish. This form provides structure for consistency and transparency in reporting. For further information on Nature Portfolio policies, see our Editorial Policies and the Editorial Policy Checklist.

Statistics

For all statistical analyses, confirm that the following items are present in the figure legend, table legend, main text, or Methods section.

n/a	Confirmed	
☐	☒	The exact sample size (*n*) for each experimental group/condition, given as a discrete number and unit of measurement
☐	☒	A statement on whether measurements were taken from distinct samples or whether the same sample was measured repeatedly
☐	☒	The statistical test(s) used AND whether they are one- or two-sided<br>*Only common tests should be described solely by name; describe more complex techniques in the Methods section.*
☒	☐	A description of all covariates tested
☒	☐	A description of any assumptions or corrections, such as tests of normality and adjustment for multiple comparisons
☐	☒	A full description of the statistical parameters including central tendency (e.g. means) or other basic estimates (e.g. regression coefficient) AND variation (e.g. standard deviation) or associated estimates of uncertainty (e.g. confidence intervals)
☐	☒	For null hypothesis testing, the test statistic (e.g. *F*, *t*, *r*) with confidence intervals, effect sizes, degrees of freedom and *P* value noted<br>*Give P values as exact values whenever suitable.*
☒	☐	For Bayesian analysis, information on the choice of priors and Markov chain Monte Carlo settings
☒	☐	For hierarchical and complex designs, identification of the appropriate level for tests and full reporting of outcomes
☒	☐	Estimates of effect sizes (e.g. Cohen's *d*, Pearson's *r*), indicating how they were calculated

Our web collection on statistics for biologists contains articles on many of the points above.

Software and code

Policy information about availability of computer code

Data collection	ChIP-Seq - The Real Time Analysis software (RTA 3.4.4) was used for processing raw data files, the Illumina bcl2fastq v2.20 was used to demultiplex and convert binary base calls and qualities to fastq format. Samples were trimmed for adapters using Cutadapt (1.18) before the alignment. The trimmed reads were aligned with mm10 reference using Bowtie2(2.2.6) alignment (see methods); ATAC-Seq - FThe Real Time Analysis software (RTA 3.9.25) was used for processing raw data files, the Illumina bcl2fastq v2.17 was used to demultiplex and convert binary base calls and qualities to fastq format. Samples were trimmed for adapters using Cutadapt (1.18) before the alignment. The trimmed reads were aligned with mm10 reference using Bowtie2(2.2.6) alignment(see method) ; bulk RNA-Seq - Reads of the samples were trimmed for adapters and low-quality bases using Cutadapt (1.18) before alignment with the reference genome (mm10) and the annotated transcripts using STAR (2.6.0c). The mapping statistics are calculated using Picard software (2.18.26). Library complexity is measured in terms of unique fragments in the mapped reads using Picard (2.18.26)'s MarkDuplicate utility. In addition, the gene expression quantification analysis was performed for all samples using STAR/RSEM (1.3.2) tools. ; scRNA-Seq - The analysis was performed with the Cell Ranger 7.1.0 software using the default parameters
Data analysis	Softwares used for data analysis of ChIP-Seq, ATAC-Seq, bulk RNA-Seq ,HiC and scRNA-Seq are described in the methods section. No custom code or algorythms were utilized.

For manuscripts utilizing custom algorithms or software that are central to the research but not yet described in published literature, software must be made available to editors and reviewers. We strongly encourage code deposition in a community repository (e.g. GitHub). See the Nature Portfolio guidelines for submitting code & software for further information.

Data

Policy information about availability of data

All manuscripts must include a data availability statement. This statement should provide the following information, where applicable:

- Accession codes, unique identifiers, or web links for publicly available datasets
- A description of any restrictions on data availability
- For clinical datasets or third party data, please ensure that the statement adheres to our policy

The datasets generated and analyzed in this publication have been deposited in NCBI's Gene Expression Omnibus (Edgar et al., 2002) and are accessible through GEO Superseries accession number GSE248038 (https://www.ncbi.nlm.nih.gov/geo/query/acc.cgi?acc=GSE248038).

Research involving human participants, their data, or biological material

Policy information about studies with human participants or human data. See also policy information about sex, gender (identity/presentation), and sexual orientation and race, ethnicity and racism.

Reporting on sex and gender	*Use the terms sex (biological attribute) and gender (shaped by social and cultural circumstances) carefully in order to avoid confusing both terms. Indicate if findings apply to only one sex or gender; describe whether sex and gender were considered in study design; whether sex and/or gender was determined based on self-reporting or assigned and methods used.*<br>*Provide in the source data disaggregated sex and gender data, where this information has been collected, and if consent has been obtained for sharing of individual-level data; provide overall numbers in this Reporting Summary. Please state if this information has not been collected.*<br>*Report sex- and gender-based analyses where performed, justify reasons for lack of sex- and gender-based analysis.*
Reporting on race, ethnicity, or other socially relevant groupings	*Please specify the socially constructed or socially relevant categorization variable(s) used in your manuscript and explain why they were used. Please note that such variables should not be used as proxies for other socially constructed/relevant variables (for example, race or ethnicity should not be used as a proxy for socioeconomic status).*<br>*Provide clear definitions of the relevant terms used, how they were provided (by the participants/respondents, the researchers, or third parties), and the method(s) used to classify people into the different categories (e.g. self-report, census or administrative data, social media data, etc.)*<br>*Please provide details about how you controlled for confounding variables in your analyses.*
Population characteristics	*Describe the covariate-relevant population characteristics of the human research participants (e.g. age, genotypic information, past and current diagnosis and treatment categories). If you filled out the behavioural & social sciences study design questions and have nothing to add here, write "See above."*
Recruitment	*Describe how participants were recruited. Outline any potential self-selection bias or other biases that may be present and how these are likely to impact results.*
Ethics oversight	*Identify the organization(s) that approved the study protocol.*

Note that full information on the approval of the study protocol must also be provided in the manuscript.

Field-specific reporting

Please select the one below that is the best fit for your research. If you are not sure, read the appropriate sections before making your selection.

- [x] Life sciences
- [ ] Behavioural & social sciences
- [ ] Ecological, evolutionary & environmental sciences

For a reference copy of the document with all sections, see nature.com/documents/nr-reporting-summary-flat.pdf

Life sciences study design

All studies must disclose on these points even when the disclosure is negative.

Sample size	ATAC-Seq, ChIP-Seq, bulk RNA-Seq were all carried out with 2-3 biological replicates. Sample sizes were limited by funding required for expensive genomics experiments.
Data exclusions	No data was excluded.
Replication	All relevant data from biological replicates are shown in the manuscript. Biological replicates were used to verify reproducibility.
Randomization	macrophages for each replicate of ChIP-Seq, ATAC-Seq ,RNA-Seq ,HiC and scRNA-seq were purified from 5-10 randomized mice.
Blinding	Experimental data were gathered in a non-blinded fashion. Blinding was not possible as the mice are visually distinct. Bioinformatic analyses were carried out without a priori knowledge of the experimental design.

Reporting for specific materials, systems and methods

We require information from authors about some types of materials, experimental systems and methods used in many studies. Here, indicate whether each material, system or method listed is relevant to your study. If you are not sure if a list item applies to your research, read the appropriate section before selecting a response.

Materials & experimental systems

n/a	Involved in the study
☐	☒ Antibodies
☒	☐ Eukaryotic cell lines
☒	☐ Palaeontology and archaeology
☐	☒ Animals and other organisms
☒	☐ Clinical data
☒	☐ Dual use research of concern
☒	☐ Plants

Methods

n/a	Involved in the study
☐	☒ ChIP-seq
☒	☐ Flow cytometry
☒	☐ MRI-based neuroimaging

Antibodies

Antibodies used	anti-H3K27ac (39133, Active Motif)
Validation	anti-H3K27ac (39133, Active Motif)has been utilized in 146 publications

Animals and other research organisms

Policy information about studies involving animals; ARRIVE guidelines recommended for reporting animal research, and Sex and Gender in Research

Laboratory animals	BALB/cJ and C57BL/6J mice, CB6F1/J (Jax no. 100007) typically 6 to 8 weeks old, were purchased from The Jackson Laboratory.
Wild animals	No wild animals were used.
Reporting on sex	the results finding apply only on male mice
Field-collected samples	No field-collected samples were used.
Ethics oversight	NIH Intramural Research Program – NIAID – LPD16E

Note that full information on the approval of the study protocol must also be provided in the manuscript.

Plants

Seed stocks	*Report on the source of all seed stocks or other plant material used. If applicable, state the seed stock centre and catalogue number. If plant specimens were collected from the field, describe the collection location, date and sampling procedures.*
Novel plant genotypes	*Describe the methods by which all novel plant genotypes were produced. This includes those generated by transgenic approaches, gene editing, chemical/radiation-based mutagenesis and hybridization. For transgenic lines, describe the transformation method, the number of independent lines analyzed and the generation upon which experiments were performed. For gene-edited lines, describe the editor used, the endogenous sequence targeted for editing, the targeting guide RNA sequence (if applicable) and how the editor was applied.*
Authentication	*Describe any authentication procedures for each seed stock used or novel genotype generated. Describe any experiments used to assess the effect of a mutation and, where applicable, how potential secondary effects (e.g. second site T-DNA insertions, mosiacism, off-target gene editing) were examined.*

ChIP-seq

Data deposition

☒ Confirm that both raw and final processed data have been deposited in a public database such as GEO.

☒ Confirm that you have deposited or provided access to graph files (e.g. BED files) for the called peaks.

Data access links<br>*May remain private before publication.*	GEO accession number GSE248023 (https://www.ncbi.nlm.nih.gov/geo/query/acc.cgi?acc=GSE248023)
Files in database submission	BALBc_IL4_H3K27ac_1.fastq.gz ,BL6_IL4_H3K27ac_1.fastq.gz,BALBc_IL4_H3K27ac_2.fastq.gz, BL6_IL4_H3K27ac_2.fastq.gz

	BALBc_input.fastq.gz , BL6_input.fastq.gz,BALBc_IL4_H3K27ac_1.sorted.bw , BL6_IL4_H3K27ac_1.sorted.bw, BALBc_IL4_H3K27ac_2.sorted.bw ,BL6_IL4_H3K27ac_2.sorted.bw,BALBc_input.sorted.bw,BL6_input.sorted.bw
Genome browser session (e.g. UCSC)	Integrative Genomics Viewer: https://igv.org

Methodology

Replicates	2 replicates for each ChIP-seq sample
Sequencing depth	All the samples have yields between 48 and 71 million pass filter reads.
Antibodies	anti-H3K27ac (39133, Active Motif);
Peak calling parameters	Peaks were called by MACS2(2.2.6) with –broad option on
Data quality	Except peak calling , we also use deepTools to see the heapmaps results of some specific regions. A PCA plot is drawn to verify the data quality.
Software	The Real Time Analysis software (RTA 3.4.4) was used for processing raw data files, the Illumina bcl2fastq v2.20 was used to demultiplex and convert binary base calls and qualities to fastq format. Samples were trimmed for adapters using Cutadapt (1.18) before the alignment. The trimmed reads were aligned with mm10 reference using Bowtie2(2.2.6) alignment. Library complexity is measured by uniquely aligned reads using picard (2.18.26)'s markduplicate utility. Peaks were called by MACS2(2.2.6) with –broad option on. HOMER (v4.10.4) findPeaks -style super was used to find super enhancers in ChIP-seq. Enrichment analysis of strain-specific enhancer regions based on biological process Gene Ontology (GO) terms was conducted using the Genomic Regions Enrichment of Annotations Tool (GREAT) (http://great.stanford.edu/). Bigwig files are generated by bamCoverage(deeptools/3.5.0), with the parameter --binSize 10 –normalize using RPGC.
